# Copper Status in Alzheimer's Disease and Other Neurodegenerative Disorders 2013

**DOI:** 10.1155/2013/838274

**Published:** 2013-12-19

**Authors:** Rosanna Squitti, Tjaard Hoogenraad, George Brewer, Ashley I. Bush, Renato Polimanti

**Affiliations:** ^1^Department of Neuroscience, AFaR-Ospedale “San Giovanni Calibita” Fatebenefratelli, Isola Tiberina, 00186 Rome, Italy; ^2^Neurodegeneration Laboratory, IRCCS San Raffaele Pisana, 00166 Rome, Italy; ^3^Department of Neurology, University Hospital Utrecht, 3584 CX Utrecht, The Netherlands; ^4^Departments of Human Genetics and Internal Medicine, University of Michigan Medical School, Ann Arbor, MI 48109, USA; ^5^Florey Institute of Neuroscience & Mental Health, University of Melbourne, Melbourne, VIC 3053, Australia; ^6^Department of Biology, University of Rome “Tor Vergata”, 00133 Rome, Italy

In recent years, the number of studies about the role of copper metabolism in the pathophysiology of Alzheimer's disease (AD) has been rapidly increasing. A wide range of experimental approaches have been used to explore this important issue: in vitro analyses, investigations on animal models, bioinformatic surveys, and patients' studies. Most of these studies have indicated that systemic disarrangements of copper metabolism can be one of the pathologic pathways at the basis of AD, and moreover, preventive and therapeutic strategies based on copper may be developed in order to slow down or to block the disease progression. However, further studies are necessary to translate these findings into clinical practice. In 2011, the International Journal of Alzheimer's Disease (IJAD) published the annual issue “*Copper Status in Alzheimer's Disease and Other Neurodegenerative Disorders*”, in order to collect some relevant studies about copper and AD. After two years, due to the growing literature about this topic, we and IJAD have decided to present the 2013 annual issue. In this issue, studies from different international authors are published about the recent advances in the field of copper metabolism in AD. We decided to include investigations based on different approaches in order to display the multiplicity of experimental methodologies, currently present to investigate this important subject.

One of the paper of this annual issue is a review article by B. G. Schreurs about the use of the AD rabbit model based on a 2% cholesterol diet to explore the role of copper in drinking water in determining the AD progression, especially highlighting the outstanding contribution of the late D. L. Sparks.

Another paper is provided by K. Singh and collaborators. They present novel data about the positive effects of a new copper chelator, L-2,6-pyridinedicarboxylic acid, 2,6-bis[2-[(4-carboxyphenyl) methylene] hydrazide], in the eye tissues of transgenic Drosophila expressing beta-amyloid (A*β*).

One of the paper is a review article by G. J. Brewer and S. Kaur about the importance of ingestion of inorganic copper (such as that of drinking water) as a possible factor of AD causation and of zinc therapy to reduce serum “free” copper levels and, consequently, to slow down cognition loss in AD patients. They also furnished novel data about a 6-month small double blind trial of a new zinc formulation on AD patients.

In another paper, A. Rembach and colleagues show their outcomes on the brain-specific alteration in copper levels of AD patients. Specifically, they observed that the decrease of copper in the human frontal cortex was confined in the soluble fractions.

Two of the papers are two research articles provided by N. Arnal and collaborators. In these papers, they display the interactions between copper and cholesterol in neurodegenerative processes of Wistar rats and the effects of copper and cholesterol on mitochondrial function in a rat model.

Another paper is a review article by X. Y. Choo and collaborators. In this review, the authors focused their attention on AD-related inflammation, highlighting recent advances on the role of copper as modulator of inflammatory processes in AD.

Finally, last but not least, there is the paper by C. Salustri and collaborators. In this research article, the authors provide novel information about the role of copper in the neurophysiology of AD. Specifically, they investigated whether copper dysfunction affects neurotransmission in AD patients, highlighting the association between copper and glutamatergic and Gabaergic transmission.

In conclusion, we provided an issue with a wide range of experimental evidence about the relevant role of copper in AD pathogenesis. Although we are still far to completely understand this important topic, the data present can help the reader to better understand the recent advance in this research field.

*Rosanna Squitti* *Rosanna Squitti* *Tjaard Hoogenraad* *Tjaard Hoogenraad* *George Brewer* *George Brewer* *Ashley I. Bush* *Ashley I. Bush* *Renato Polimanti* *Renato Polimanti*

